# An RNA isolation system for plant tissues rich in secondary metabolites

**DOI:** 10.1186/1756-0500-4-85

**Published:** 2011-03-28

**Authors:** Sanjay Ghawana, Asosii Paul, Hitesh Kumar, Arun Kumar, Harsharan Singh, Pardeep K Bhardwaj, Arti Rani, Ravi S Singh, Jyoti Raizada, Kashmir Singh, Sanjay Kumar

**Affiliations:** 1 Biotechnology Division, Institute of Himalayan Bioresource Technology (CSIR), Palampur-176 061, Himachal Pradesh, India; 2 National Institute of Plant Genome Research, Aruna Asaf Ali Marg, P.O. Box No. 10531, New Delhi- 110 067, India; 3 Assistant Professor, Department of Botany, SCVB Government College, Palampur-176 061, Himachal Pradesh, India; 4 Assistant Professor, Biotechnology Division, Lyallpur Khalsa College, Jalandhar-144 001, Punjab, India; 5 Scientist, Regional Centre of Institute of Bioresources and Sustainable Development (DBT), Tadong-737 102, Sikkim, India; 6 Scientist, Vittal Mallya Scientific Research Foundation, #94/3 & 94/5, 23rd cross, 29th main, BTM II Stage, Bangalore-560 076, Karnataka, India; 7 Assistant Professor, Department of Biotechnology, Panjab University, Chandigarh-160 014, India

## Abstract

**Background:**

Secondary metabolites are reported to interfere with the isolation of RNA particularly with the recipes that use guanidinium-based salt. Such interference was observed in isolation of RNA with medicinal plants rheum (*Rheum australe*) and arnebia (*Arnebia euchroma*). A rapid and less cumbersome system for isolation of RNA was essential to facilitate any study related to gene expression.

**Findings:**

An RNA isolation system free of guanidinium salt was developed that successfully isolated RNA from rheum and arnebia. The method took about 45 min and was successfully evaluated on twenty one tissues with varied secondary metabolites. The *A*_260/280 _ratio ranged between 1.8 - 2.0 with distinct 28 S and 18 S rRNA bands visible on a formaldehyde-agarose gel.

**Conclusions:**

The present manuscript describes a rapid protocol for isolation of RNA, which works well with all the tissues examined so far. The remarkable feature was the success in isolation of RNA with those tissues, wherein the most commonly used methods failed. Isolated RNA was amenable to downstream applications such as reverse transcription-polymerase chain reaction (RT-PCR), differential display (DD), suppression subtractive hybridization (SSH) library construction, and northern hybridization.

## Background

Deciphering the underlying mechanisms of gene expression, signal transduction, gene regulation and transcriptome analysis requires a whole gamut of techniques such as northern hybridization, reverse transcription-polymerase chain reaction (RT-PCR), and construction of cDNA libraries. Substantially pure and un-degraded RNA is a fundamental requisite for all these techniques. A large number of protocols have been developed or extensively modified [1-7], and commercial kits are also available for isolation of RNA from plant tissues. Most of these methods, including kits, were found to be unsuitable for isolation of RNA from *Litchi chinensis*, *Pinus taeda*, *Pseudotsuga menziesii*, *Picea glauca*, *Griffonia simplicifolia *and *Albizia procera *[2-4,6]. Some of these tissues have phenolic compounds, which get oxidized to form quinones. Quinones bind to RNA and hinder RNA isolation and/or downstream applications [8]. Secondary metabolites often co-precipitate with RNA and affect yield, quality [3] and interfere with downstream applications [9]. Specific protocols developed for such tissues are usually time consuming and also tissue specific [2-7].

**Figure 1:**
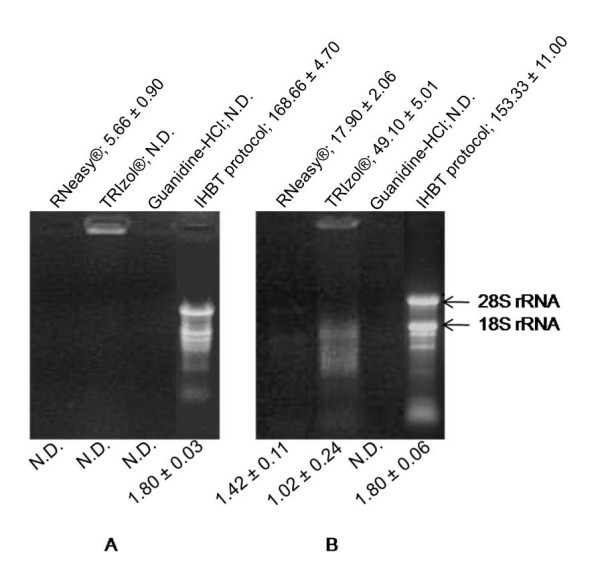
**Denaturing gel electrophoresis of RNA isolated from leaf tissues of rheum (A), and arnebia (B) using different RNA isolation methods viz. RNeasy^®^, TRIzol^®^, Guanidine-HCl, and IHBT protocol**. RNA yield (μg/100 mg tissue) is mentioned above each panel, whereas *A*_260/280 _ratio is mentioned at the bottom of the panel. To ease comparison, equal volume of RNA (2 μl) was loaded. The starting material and the volume of the DEPC-treated water used to dissolve RNA were kept same in all the four procedures. N.D., not detectable.

Our research work involved cloning of relevant genes from medicinal plants, rheum (*Rheum australe*) and arnebia (*Arnebia euchroma*), which are rich in secondary metabolites anthraquinones and alkannins/shikonins, respectively. The prevalent methods namely TRIzol^® ^(Invitrogen, USA), RNeasy^® ^(Qiagen, Germany) and guanidinium salt based method [5] either failed to isolate RNA or yielded negligible quantity of RNA from these plants (Figure 1). Three methods we tried utilized a guanidinium-based salt as one of the constituents of the RNA isolation system [1,5,10]. A guanidinium-based salt is a strong protein denaturant and inhibitor of RNase. Therefore, it is an ingredient of choice in most of the RNA isolation systems. However several tissues, including those mentioned above were recalcitrant to RNA isolation, possibly due to the presence of guanidinium salts [3,6,7]. The presence of secondary metabolites has been attributed to interfere with resuspension of RNA when extracted with guanidinium salts [3]. Since guanidinium salts are also ineffective in dissociating RNA from non-protein complexes, RNA may be lost along with the complex during isolation procedure [11,12]. It is also likely that the presence of guanidinium salt might promote such a complex formation that would further inhibit RNA isolation. Therefore, it was necessary to develop a composition which is free of guanidinium-based salt and at the same time the composition should possess protein-denaturant activity strong enough to inhibit RNase action.

Compositions free of guanidinium salt have been developed for isolation of RNA [3,6,7]. However, these procedures are time consuming, cumbersome, expensive and hence limit simultaneous processing of large number of samples. The present manuscript describes a protocol (IHBT protocol) for isolation of RNA from rheum and arnebia, which does not utilize guanidinium salt and also is simple and rapid. The developed protocol was extended to nineteen more plant tissues with success and the RNA isolated was amenable to downstream applications.

## Methods

A total of 21 plant tissues were collected either from (i) the wild, (ii) cultivation in the experimental farm of the institute, or (iii) the local market.

### (a) Solutions and reagents

• Water was always treated with 0.1% (v/v) diethyl pyrocarbonate (DEPC) following standard procedure as detailed by Sambrook *et al*. [10].

• Solution I: phenol saturated with tris(hydroxymethyl)aminomethane buffer to a pH of 6.7 ± 0.2 was procured from Sigma, USA (catalogue number P4557). To this was added sodium dodecyl sulphate [SDS; 0.1% (w/v)], sodium acetate [NaOAc; 0.32 M (w/v)] and ethylenediaminetetra acetic acid (EDTA; 0.01 M final concentration from a stock solution of 0.5 M, pH 8.0).

• Chloroform

• 70% Ethanol

• Isopropanol

### (b) Protocol for Isolation of RNA

i. Grind 10-100 mg of tissue to a fine powder in liquid nitrogen using a mortar and pestle.

ii. Add 2 ml of solution I, grind further. Solution I gets frozen as added; make fine powder of the frozen material; continue grinding so as to make a homogenous mixture; this ensures close contact of the tissue ingredients and the reagents that would help in instantaneous denaturation of protein. Allow to thaw completely with intermittent grinding.

iii. Add 800 μl of DEPC-treated water, and mix it by grinding.

iv. Transfer the contents to two, 2 ml micro-centrifuge tubes and leave for 5 min at room temperature.

v. Add 200 μl of chloroform to each tube, vortex briefly (< 10 s) and leave for 10 min at room temperature.

vi. Centrifuge at 13,000 rpm for 10 min at 4 °C and transfer the upper aqueous phase into fresh tubes.

vii. Add 0.6 volumes of isopropanol, vortex briefly (< 10 s) and leave for 10 min at room temperature.

viii. Centrifuge at 13,000 rpm for 10 min at 4 °C and discard the supernatant.

ix. Wash RNA pellet with 70% ethanol, air dry and dissolve in 20 to 50 μl of DEPC-treated water.

Purity and concentration of RNA was assessed by determining the absorbance of the sample at 260 and 280 nm using a spectrophotometer (Specord 200, Analytica Jena, AG, Germany). Integrity of RNA was evaluated on a denaturing formaldehyde-agarose gel as described previously [13]. First strand cDNA was synthesized after digestion of RNA with RNase-free DNase I (amplification grade, Invitrogen, USA) and used for the amplification of *26 S rRNA *[14]. Isolated RNA was also used for DD, northern analysis, SSH and rapid amplification of cDNA ends (RACE), and published elsewhere [13,15-17].

## Results and discussion

A phenol-based, guanidinium salt-free protocol for isolation of RNA was developed. Reagents were selected based on their specific properties: phenol as a strong protein denaturant and inhibitor of RNase; SDS and EDTA are also inhibitors of RNase [18]. This composition provided a cocktail of RNase inhibitors and protein denaturant. Further, pH of the solution was maintained in the acidic range to allow efficient and preferable partitioning of RNA in the aqueous phase leaving DNA in the phenolic phase; DNA prefers basic pH for its partitioning into the aqueous phase [19]. An appropriate concentration of NaOAc was included in the solution to aid precipitation of RNA in the presence of isopropanol. The addition of DEPC-treated autoclaved water after addition of solution I rendered sufficient aqueous environment for partitioning of RNA into the aqueous phase. Our results suggested that the composition developed in the present communication had requisite protein denaturation and RNase inhibitory activity. It appears that the composition and the protocol provided an environment which prevented oxidation of phenolics leading to quick removal of these compounds from the extraction medium. This possibly allowed RNA to be free from quinone/protein complexes, and hence eased extraction and further re-suspension in water.

**Figure 2:**
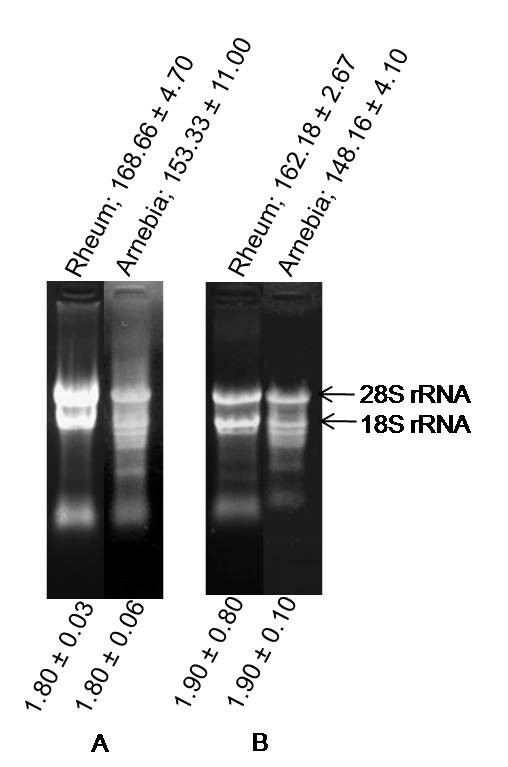
**Denaturing gel electrophoresis of RNA isolated from leaf tissues of rheum and arnebia**: (A) before treatment with DNase, and (B) after treatment with DNase as detailed in the Methods section. RNA yield (μg/100 mg tissue) is mentioned above each panel, and *A*_260/280 _ratio is mentioned at the bottom of the panel. Clear 28 S and 18 S rRNA bands show integrity of the RNA.

The developed method successfully isolated RNA from leaf tissues of rheum and arnebia with a yield of 168.66 ± 4.7 and 153.33 ± 11 μg RNA/100 mg of tissue, respectively. This was remarkable in the light that other protocols either failed to yield RNA or yielded extremely low quantity (Figure 1). Though contamination of DNA can not be completely avoided, as is also reported with most of the rapid protocols [20,21], digestion of RNA with RNase-free DNase took care of the contamination (Figure 2). In fact, DNase digestion is a common step in protocols involving RNA analysis [13-15].

**Figure 3:**
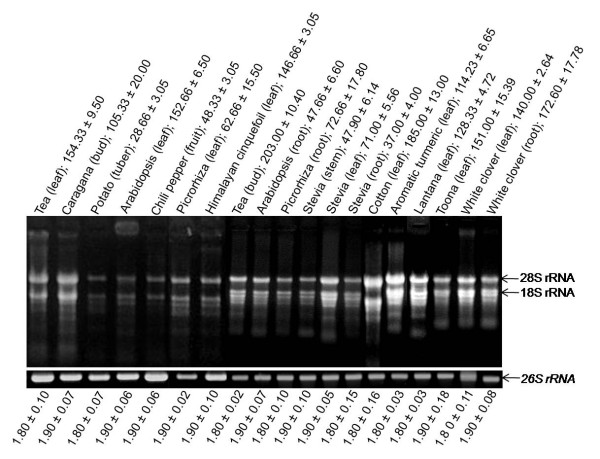
**Denaturing gel electrophoresis of RNA isolated from different plant tissues using IHBT protocol**. Name of plant tissues along with RNA yield (μg/100 mg tissue) is written above each panel; *A*_260/280 _ratio is mentioned at the bottom of the panel. The quality was assessed by electrophoresing an equal volume of RNA (2 μl) on formaldehyde-agarose denaturing gel to observe integrity of 28 S and 18 S rRNA bands, and by confirming the amenability of isolated RNA to reverse transcription-polymerase chain reaction based amplification of *26 S rRNA*. Tea (*Camellia sinensis*, Family: Theaceae), potato (*Solanum tuberosum*, Family: Solanaceae), chili pepper (*Capsicum annum*, Family: Solanaceae), picrorhiza (*Picrorhiza kurrooa*, Family: Scrophulariaceae), stevia (*Stevia rebaudiana*, Family: Asteraceae), aromatic turmeric (*Curcuma aromatica*, Family: Zingiberaceae), and lantana (*Lantana camara*, Family: Verbenaceae) is rich in flavanoids, polysaccharides, capsaicinoids, picrosides, steviosides, curcuminoids, and triterpenoids, respectively. Arabidopsis (*Arabidopsis thaliana*, Family: Brassicaceae) is a model plant, whereas the knowledge on the nature of secondary metabolites in caragana (*Caragana jubata*, Family: Fabaceae), Himalayan cinquefoil (*Potentilla atrosanguinea*, Family: Rosaceae), cotton (*Gossypium hirsutum*, Family: Malvaceae), toona (*Toona sinensis*, Family: Meliaceae), and white clover (*Trifolium repens*, Family: Fabaceae) was not available.

The method so developed was further extended to nineteen more tissues (Figure 3) from different plants having varying amount of secondary metabolites. The *A*_260/280 _ratio of the isolated RNA ranged between 1.8 - 2.0, which indicated the RNA to be relatively free of proteins and contaminants. At times, low *A*_260/280 _ratio ranging between 1.4 - 1.7 was also obtained, particularly when higher amount of the tissue was used. In all the cases, distinct 28 S and 18 S rRNA bands were observed on 1% formaldehyde-agarose denaturing gel indicating intact RNA. Also, the isolated RNA was amenable to downstream applications e.g. RT-PCR, DD, northern analysis, SSH and RACE, as reported in our publications that utilized the present protocol [13,15-17,22,23].

## Conclusion

Unlike specific protocols for different tissues, the developed method was very useful in isolation of RNA not only from rheum and arnebia but also from various other plant species belonging to diverse genera. The distinguishing feature of the protocol was the success with those tissues wherein the commonly used protocols failed. The protocol is simple, does not require any specialized material, chemical, instrument and procedure such as ultracentrifugation step through cesium chloride gradient or lithium chloride precipitation, thereby greatly reducing the complications and the time required.

## Competing interests

The authors declare that they have no competing interests.

## Authors' contributions

SG standardized various solutions and optimized protocol. AP, HK, AK, HS, PKB, AR, RSS, JR and KS carried out the experiments and participated in manuscript writing along with SG. SK guided on development of the RNA isolation system, guided the research and finally drafted the manuscript. All the authors have read the manuscript and agree with the content.
